# Integrated transcriptomic analysis reveals immune signatures distinguishing persistent versus resolving outcomes in MRSA bacteremia

**DOI:** 10.3389/fimmu.2024.1373553

**Published:** 2024-05-23

**Authors:** Rajesh Parmar, Harry Pickering, Richard Ahn, Maura Rossetti, David W. Gjertson, Felicia Ruffin, Liana C. Chan, Vance G. Fowler, Michael R. Yeaman, Elaine F. Reed, MRSA Systems Immunology Group, Rajesh Parmar

**Affiliations:** ^1^ Department of Pathology and Laboratory Medicine, David Geffen School of Medicine, University of California Los Angeles, Los Angeles, CA, United States; ^2^ Department of Microbiology, Immunology, & Molecular Genetics, University of California Los Angeles, Los Angeles, CA, United States; ^3^ Division of Infectious Diseases, Duke University, Durham, NC, United States; ^4^ Department of Medicine, David Geffen School of Medicine, University of California Los Angeles, Los Angeles, CA, United States; ^5^ Divisions of Molecular Medicine and Infectious Diseases, Los Angeles County Harbor-UCLA Medical Center, Torrance, CA, United States; ^6^ Lundquist Institute for Biomedical Innovation at Harbor-UCLA Medical Center, Torrance, CA, United States

**Keywords:** *Staphylococcus aureus*, MRSA, persistence, transcriptomics, proteomics, host immunity

## Abstract

**Introduction:**

*Staphylococcus aureus* bacteremia (SAB) is a life-threatening infection particularly involving methicillin-resistant *S. aureus* (MRSA). In contrast to resolving MRSA bacteremia (RB), persistent MRSA bacteremia (PB) blood cultures remain positive despite appropriate antibiotic treatment. Host immune responses distinguishing PB vs. RB outcomes are poorly understood. Here, integrated transcriptomic, IL-10 cytokine levels, and genomic analyses sought to identify signatures differentiating PB vs. RB outcomes.

**Methods:**

Whole-blood transcriptomes of propensity-matched PB (n=28) versus RB (n=30) patients treated with vancomycin were compared in one independent training patient cohort. Gene expression (GE) modules were analyzed and prioritized relative to host IL-10 cytokine levels and DNA methyltransferase-3A (*DNMT3A*) genotype.

**Results:**

Differential expression of T and B lymphocyte gene expression early in MRSA bacteremia discriminated RB from PB outcomes. Significant increases in effector T and B cell signaling pathways correlated with RB, lower IL-10 cytokine levels and *DNMT3A* heterozygous A/C genotype. Importantly, a second PB and RB patient cohort analyzed in a masked manner demonstrated high predictive accuracy of differential signatures.

**Discussion:**

Collectively, the present findings indicate that human PB involves dysregulated immunity characterized by impaired T and B cell responses associated with excessive IL-10 expression in context of the *DNMT3A* A/A genotype. These findings reveal distinct immunologic programs in PB vs. RB outcomes, enable future studies to define mechanisms by which host and/or pathogen drive differential signatures and may accelerate prediction of PB outcomes. Such prognostic assessment of host risk could significantly enhance early anti-infective interventions to avert PB and improve patient outcomes.

## 1 Introduction

*Staphylococcus aureus* bacteremia (SAB) represents a common, life-threatening and emerging bloodstream infection (1, 2) accounting for up to 15% of hospital-acquired infections in the United States (3, 4) and greater frequency worldwide. Among these cases, antibiotic-persistent *S. aureus* bacteremia is of urgent and growing concern. This condition occurs when the infecting clinical isolate of *S. aureus* is not cleared from the bloodstream, despite appropriate dosing and pharmacology of anti-staphylococcal therapy to which the isolate is susceptible *in vitro* per CLSI breakpoints. Such persistent bacteremia cases are prevalent and potentially lethal (5–7), particularly when involving methicillin-resistant *S. aureus* (MRSA). The etiologies of SAB are diverse, including invasive skin/soft tissue infections, catheter-associated infections, prosthetic joint infections, and endocarditis among others (7–12). In patients with MRSA bacteremia, outcomes vary greatly depending on the source and hematogenous dissemination of infection (13). Antibiotic-persistent MRSA bacteremia (PB) occurs when the infecting isolate is not cleared from the bloodstream despite appropriate treatment with an antibiotic to which it exhibits susceptibility *in vitro.* This paradox suggests that differential host responses in context of anti-infective therapy contribute to clinical outcomes in the face of SAB due to a particular MRSA isolate (14).

Specific host-pathogen interactions influencing outcomes of MRSA infection have been a topic of increasing research (15, 16). Yet, the intersection of host and *S. aureus* mechanisms underlying antibiotic persistent vs. resolution of MRSA bacteremia (RB) remain incompletely understood. In recent years, development of high-throughput genomic, transcriptomic and proteomic platforms has enabled identification of disease-associated immune phenotypes (17, 18). In this study, we tested the hypothesis that peripheral blood transcriptional profiling integrated with proteomic and genotypic correlates would offer new insights into immune mechanisms impacting PB vs. RB outcomes in MRSA bacteremia.

There were four explicit goals of the current investigation: 1) identify transcriptional signatures that differentiate PB vs. RB outcomes in the setting of gold-standard vancomycin therapy in the absence of other omics data; 2) discern relationships linking transcriptomic, IL-10 cytokine levels and genotypic signatures to further enhance differential host response signatures in such PB vs. RB outcomes; 3) disclose putative molecular and cellular mechanisms that may impact these differential outcomes; and 4) evaluate the predictive accuracy of signatures identified using a separate cohort of patients with PB or RB outcomes. Patients having PB vs. RB outcomes exhibited significantly differential patterns of gene co-expression. Specifically, up-regulation of T and B cell signaling genes were hallmarks of RB outcomes, particularly when integrated with low IL-10 levels and host *DNMT3A* A/C genotype associated with resolution. These findings suggest host transcriptional responses in context of genotypic regulation may shape cellular and proteomic host responses necessary for clearing of MRSA infection in the setting of vancomycin treatment. Such findings further substantiate the potential for systems immunology applications to enhance predictive, diagnostic, or prognostic assessment that could guide medical intervention for improved clinical outcomes.

## 2 Materials and methods

### 2.1 Study cohort

This case-controlled study consisted of 85 SAB patients from two cohorts. Cohort-1 consisted of 58 patients (28 PB and 30 RB) propensity matched by sex, race, age, hemodialysis status, type I diabetes, or presence of an implantable device. Details of clinical characteristics of study cohort-1 are presented in **Table 1**. In general, patients with persistent bacteremia (PB = 28) had higher rates of endovascular sources of infection (PB = 7 and RB = 2), metastatic infection (n=33) including metastatic endocarditis (PB = 7 and RB = 2), metastatic vertebral osteomyelitis (PB = 5 and RB = 1), metastatic nonvertebral osteomyelitis (PB = 4 and RB = 2), longer length of stay (PB = 20.5 days and RB = 13.1 days), and worse overall outcomes compared to RB (n=30). Cohort-2 was a separate validation cohort that consisted of 27 patients (13 PB and 14 RB), where the clinical data and clinical outcomes were blinded to the investigators. SAB cases were evaluated and consented at enrolment in the *S. aureus* Bacteremia Group (SABG) biorepository at Duke University Medical Centre (DUMC). Cases for the current study were carefully selected based on the following inclusion criteria: laboratory confirmed MRSA bacteremia; receipt of broad-spectrum antibiotic therapy that included vancomycin for suspected bacteremia due to high prevalence of MRSA; vancomycin administration based on therapeutic drug level monitoring for all patients; enrolled in the SABG study between 2007 and 2017 (to ensure contemporary medical practices). Clinical PB was defined as any patient having continuous MRSA positive blood cultures for at least 5 days after vancomycin antibiotic treatment (7). Clinical RB was defined as any patient having negative blood cultures within 5 days after the initiation of vancomycin therapy. The duration of therapy varied based on the extent of the infection, but generally ranged from two to six weeks.

**Table 1:** Characteristics of the MRSA study cohort: clinical and laboratory parameters.

Characteristic		Cohorts (cohort-1 N = 58)		
		Persistent Bacteremia (PB) (n=28)	Resolving Bacteremia (RB) (n=30)	p-value
Demographics				
	Age (years, mean ± SD)	64.10 ± 13.9	62.64 ± 12.6	0.998
	Gender (male/female)	19/9	21/9	0.720
Race				
	Black	13	14	1.000
	Caucasian	15	15	1.000
	Unknown	0	1	1.000
Underlying comorbidity				
	Neoplasm	0	7	0.009
	Diabetic	14	18	0.825
	Hemodialysis dependent	13	9	0.177
	HIV positive	1	0	1.000
	Transplant Recipient	1	5	0.060
	Injection Drug Use	1	0	1.000
	Corticosteroid Use (30 day)	7	7	1.000
	Surgery Past 30 Days	5	8	0.276
	Endocarditis, previous episodes	1	1	1.000
Site of Acquisition				
	Hospital-acquired	1	3	0.627
	HCA community -acquired	25	24	1.000
	Non-HCA community -acquired	2	3	1.000
Source of Bacteremia				
	Endovascular infection	7	2	0.153
	GI/GU infection	4	5	1.000
	Respiratory/Lung	2	1	0.612
	Skin, soft tissue, joint/bone infection	7	8	1.000
	None/Unknown	4	7	0.526
Implantable devices				
	Heart Valve	1	0	1.000
	Joint	3	1	1.000
	Orthopedic rod	0	0	NA
	Plate and Screw	3	1	1.000
	Bone Plate	0	0	NA
	Intravascular graft	0	2	0.154
	Hemodialysis graft	8	3	0.366
	Pacemaker/defibrillator	8	6	0.661
	Indwelling intravascular catheter	7	4	0.575
Metastatic infection		19	14	0.317
	Metastatic endocarditis	7	2	0.097
	Metastatic vertebral osteomyelitis	5	1	0.063
	Metastatic nonvertebral osteomyelitis	4	2	0.554
Average length of stay		20.5 days	13.1 days	NA
Duration of antibiotics, days (mean ± SD)		51.0 ± 18.5	30.5 ± 17.5	< 0.001
Host *DNMT3A* genotype				
	Heterozygous A/C	3	18	<0.001
	Homozygous A/A	25	12	0.001

MRSA (Methicillin-Resistant Staphylococcus aureus), PB (Antibiotic-Persistent MRSA bacteremia), RB (Antibiotic-Resolving MRSA bacteremia), HCA, Healthcare-associated; GI/GU, Gastrointestinal/Genitourinary.

PB and RB subjects were considered eligible for inclusion if they were successfully matched 1:1 by sex, age, race, hemodialysis status, diabetes mellitus, and presence of any implantable medical device using nearest neighbor propensity scores generated from logistic regression models fit separately across 4 strata (**Supplementary Table S1**) (19). Empiric vancomycin therapy was initiated in all patients in each cohort prior to blood draw for analysis. Peripheral blood transcriptome profile and cytokine IL-10 levels were obtained from vancomycin-treated MRSA bacteremia patients collected at the time of initial diagnosis.

### 2.2 IL-10 cytokine profiling

We utilized Human 38-plex magnetic cytokine/chemokine kits (EMD Millipore, HCYTMAG-60K-PX38) per manufacturer instructions. For quantification serum IL-10, we employed a Luminex 200TM instrument, and concentrations of each analyte were computed using Milliplex Analyst software version 4.2 (EMD Millipore). The Luminex assay and analysis were conducted by the UCLA Immune Assessment Core.

### 2.3 RNA sequencing, mapping, quantifications, and quality control

Total RNA was isolated with Qiagen RNA Blood kit, and QC was performed with Nanodrop 8000 and Agilent Bioanalyzer 2100. Globin RNA was removed with Life Technologies GLOBINCLEAR (human) kit. Libraries for RNA-seq were prepared with KAPA Stranded RNA-seq Kit. The workflow consists of mRNA enrichment, cDNA generation, and end repair to generate blunt ends, A-tailing, adaptor ligation and PCR amplification. Different adaptors were used for multiplexing samples in one lane. Sequencing was performed on Illumina Hiseq3000 for a single read 50 run. Each sample generated an average of 15 million reads. Data quality check was done on Illumina SAV. Demultiplexing was performed with Illumina Bcl2fastq2 v 2.17 program.

### 2.4 Weighted gene co-expression analysis (WGCNA)

In our study, we used R package ‘WGCNA’ to construct a gene co-expression network (20). Before performing the WGCNA, we used normalization with 12720 genes and selected the top 5000 expressed genes. After filtering out the low expressed genes, normalized expression data were transformed by using the voom transformation method (21, 22) using the integrated function in the WGCNA package. After filtering out the low expressed genes in the dataset, the next step of WGCNA is to build a scale-free network. In a scale-free network, several nodes, which are called hub nodes, are highly connected to other nodes in the network (22). In our study, we use the unsigned co-expression measure, which means that the positive correlation and negative correlation are equal. We constructed the gene co-expression network using the following steps.

First, a soft thresholding power (β) to which co-expression similarity was raised to calculate adjacency. By raising the absolute value of the correlation to a power β ≥ 1 (soft thresholding), the weighted gene co-expression network construction emphasizes high correlations at the expense of low correlations. To determine the best soft threshold power, scale independence and average connectivity degree of modules with different power values were calculated by the gradient method. We selected the power β to ensure that the co-expression network was a ‘scale-free’ network, which was biologically close to reality (R^2^ > 0.9). Moreover, to minimize the effects of noise and spurious associations, we subsequently constructed the Topology Overlap Matrix (TOM) from the adjacency matrix and calculated the corresponding dissimilarity matrix (1-TOM).

### 2.5 Identification of co-expression modules

In WGCNA, we used the dynamic tree cut method to hierarchically cluster genes using the dissimilarity matrix (1-TOM) (23). The minimum size of a module was set as 30 genes, and modules with high similarity were identified by clustering and then merged with a height cut-off of 0.98.

### 2.6 Identification of modules associated with clinical trait of MRSA infection

The module eigengenes (MEs), which were measured by principal component analysis (PCA), were generated for each GE module along with the module identification procedure (24). We used MEs as a representative of the gene expression profiles in each GE modules, with logistic regression analysis to identify modules of highest interest. Next, we performed a module-trait relationship analysis by calculating the correlation between module eigengenes, *DNMT3A* genotype, and clinical outcome of MRSA infection.

### 2.7 Identification of hub genes in PPI networks of GE modules

The online database STRING (http://string-db.org) (25) was used to develop protein-protein interaction (PPI) networks. Cytoscape software was used to construct a PPI network and analyze the interactions of the different genes in the gene-expression (GE) modules (26). Hub genes are defined as genes with high correlation in PPI network created by using significant GE modules. Higher connectivity of genes in the PPI network means the higher probability of these genes as a key modulator in the pathway. The cytoHubba plug-in was used to screen modules of the PPI network in Cytoscape to identify the top hub genes in the network (27, 28). The PPI network was visualized with Cytoscape followed by the identification of hub genes with the maximal clique centrality (MCC) algorithm (29). Gene ontology (GO) analysis was performed to find out the functional role of significant GE modules common among all data sets using ShinyGO online servers that are based on DAVID Gene enrichment analysis tool (30).

### 2.8 Random forest predictions

Prediction of clinical outcome and calculation of a variable importance score based on the T cell and B cell hub genes data from RNA-seq data and IL-10 cytokine was performed using a random forest machine learning R-package (66). PB and RB status was predicted based using hub gene expression and IL-10 cytokine levels in ten-fold cross-validation per iteration. Then, the classifiers were trained and tested using a 10-fold cross-validation strategy. Receiver operating characteristics (ROCs) were used to estimate the sensitivity and specificity of the PB and RB classification method. The Area Under the ROC Curve (AUC) was calculated for each ROC to evaluate the accuracy of PB and RB classification. Subsequently, we used the random forest model to make predictions on the masked validation data. Briefly, we used predict function for making predictions on a blinded cohort using a trained random forest model in R (31).

### 2.9 Relationship between clinical outcomes and integrated transcriptomic signatures

To compare *DNMT3A* genotype, gene expression levels or IL-10 cytokine level between clusters of patients, binomial logistic regression was used, with dichotomized cluster membership as the dependent variable. To compare gene-module expression levels between clusters, linear regression was used, with gene-module eigen values as the dependent variable.

## 3 Results

### 3.1 Network construction and gene expression module classification

RNA-seq was performed on whole blood from 28 PB and 30 RB subjects extensively matched by sex, race, age, hemodialysis status, Type I diabetes, and presence of an implantable device (**Table 1**). WGCNA was used to identify modules of co-expressed genes associated with the clinical RB or PB phenotype. WCGNA was executed on the top 5000 expressed genes (**Supplementary Table S2**) using soft–thresholding powers ranging from 1 to 20. When the power value was set at 5, the connectivity between genes in the network satisfied the scale-free network distribution (**Supplementary Figures S1A, B**). Thus, we transformed the co-expression similarity matrix into an adjacency matrix using a soft-threshold power of 5. A hierarchical clustering tree identified 58 gene modules (GE) with correlation greater than 0.98.

### 3.2 Identification of gene modules associated with PB and RB clinical outcomes

**Figure 1:**
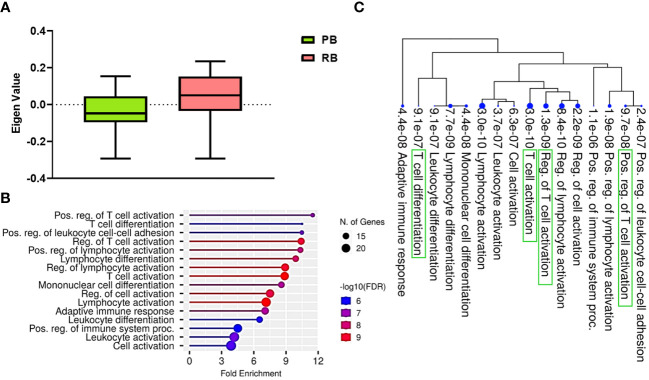
T cell signaling pathways distinguish persistent from resolving MRSA bacteremia. **(A)** Module trait relationship between ME2 and clinical outcome. Module eigengene expression distributions for RB or PB. Eigengene expression values for 28 (PB) and 30 (RB) libraries were plotted for ME2 by outcome of MRSA infection (p<0.05). **(B)** Functional characteristic analysis of ME2 gene expression. Pathway analysis was performed using the 92 differentially coexpressed ME2 genes associated with clinical outcome of MRSA infection. **(C)** Enriched biological processes of ME2. Visualization of the relationship among enriched GO categories using hierarchical clustering tree. Biological processes with shared genes are clustered together. Dot sizes are proportional to respective increasingly significant p-values. The green boxes highlight key T cell signaling pathways in the enriched gene-expression module.

ME values, which represent average expression of each gene module (GE), were assessed to identify the relationship between GE modules and PB vs. RB MRSA clinical outcomes. Logistic regression was used to identify GE modules significantly associated with PB vs. RB clinical outcome (p<0.05). GE module-2 (ME2) was significantly upregulated in RB compared to PB (**Figure 1A**) and comprised 99 genes (**Supplementary Figure S2**, **Supplementary Table S3**). To understand the function of the genes in ME2, pathway enrichment analysis was performed using the ShinyGO online tool. The top 10 enriched pathways in ME2 revealed several key biological processes principally regulating T cell and leukocyte activation and differentiation, cell-cell adhesion, adaptive immune response, and immune system processes (**Figure 1B**, **Table 2**). Significantly enriched biological processes are highlighted as an interactive clustering tree (**Figure 1C**). Key genes upregulated in ME2 as a correlate of RB include those involved in T cell signaling, such as *TCF-7*, *CD5*, *ZAP70, CD27, LCK, CD3E, IL2RB*, and *GATA3*.

**Table 2:** Pathway enrichment analysis of GE module ME2 (Top ten enriched pathways are shown).

Enrichment FDR	nGenes	Pathway Genes	Fold Enrichment	Pathway	Genes
9.67E-08	12	280	11.49378151	Pos. reg. of T cell activation	*CD6, SIRPG, GATA3, CD5, ZAP70, SLAMF1, CD27, ICOS, LCK, CARD11, CD3E, HLA-DPB1*
9.11E-07	11	281	10.49847184	T cell differentiation	*TCF7, GATA3, ZAP70, CD27, RORC, CAMK4, METTL3, GPR183, LCK, CARD11, CD3E*
2.36E-07	12	307	10.48292776	Pos. reg. of leukocyte cell-cell adhesion	*CD6, SIRPG, GATA3, CD5, ZAP70, SLAMF1, CD27, ICOS, LCK, CARD11, CD3E, HLA-DPB1*
1.28E-09	16	412	10.4150771	Reg. of T cell activation	*CD6, TCF7, SIRPG, GATA3, CD5, ZAP70, SLAMF1, SIT1, CD27, CAMK4, ICOS, METTL3, LCK, CARD11, CD3E, HLA-DPB1*
1.88E-08	14	363	10.34334792	Pos. reg. of lymphocyte activation	*CD6, SIRPG, GATA3, CD5, ZAP70, SLAMF1, PCID2, CD27, ICOS, GPR183, LCK, CARD11, CD3E, HLA-DPB1*
7.72E-09	15	406	9.908432338	Lymphocyte differentiation	*ITM2A, TCF7, GATA3, ZAP70, SLAMF1, PCID2, DOCK10, CD27, RORC, CAMK4, METTL3, GPR183, LCK, CARD11, CD3E*
8.35E-10	18	541	8.923083614	Reg. of lymphocyte activation	*CD6, TCF7, SIRPG, GATA3, CD5, ZAP70, SLAMF1, PCID2, SIT1, CD27, CAMK4, ICOS, METTL3, GPR183, LCK, CARD11, CD3E, HLA-DPB1*
2.96E-10	19	574	8.877310924	T cell activation	*CD6, TCF7, SIRPG, GATA3, CD5, ZAP70, SLAMF1, SIT1, CD27, CAMK4, ICOS, METTL3, NLRC3, GPR183, LCK, CARD11, CD3E, HLA-DPB1*
4.44E-08	15	469	8.57744889	Mononuclear cell differentiation	*ITM2A, TCF7, GATA3, ZAP70, SLAMF1, PCID2, DOCK10, CD27, RORC, CAMK4, METTL3, GPR183, LCK, CARD11, CD3E*
2.19E-09	19	678	7.515599514	Reg. of cell activation	*CD6, TXK, TCF7, SIRPG, GATA3, CD5, ZAP70, SLAMF1, PCID2, SIT1, CD27, CAMK4, ICOS, METTL3, GPR183, LCK, CARD11, CD3E, HLA-DPB1*

### 3.3 Identification of transcriptional modules associated with host *DNMT3A* genotype

**Figure 2:**
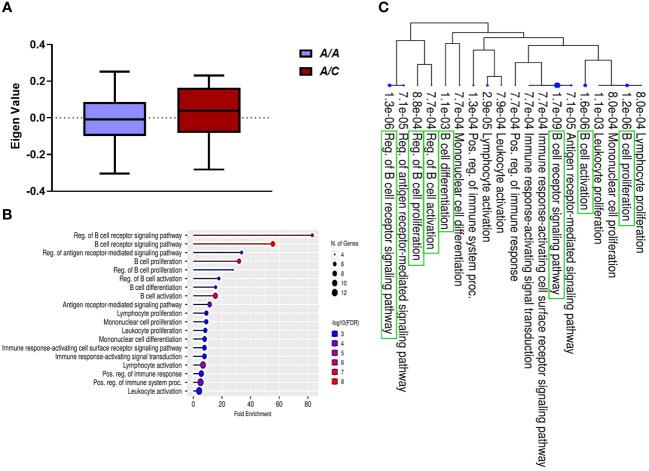
B cell signaling pathways are upregulated in patients with the *DNMT3A* A/C genotype. **(A)** Module trait relationship between ME7 with host *DNMT3A* genotype. Module eigengene expression distributions for RB or PB. Eigengene expression values for 28 (PB) and 30 (RB) libraries were plotted for ME7 by host *DNMT3A* genotype (p<0.05). **(B)** Functional characteristic analysis of ME7 gene expression. Pathway analysis was performed using the 59 differentially co-expressed ME7 genes associated with host *DNMT3A* genotype. **(C)** Enriched biological processes of ME7. Visualization of the relationship among enriched GO categories using hierarchical clustering tree. Biological processes with shared genes are clustered together. Dot sizes are proportional to respective increasingly significant p-values. The green boxes highlight key B cell signaling pathways in the enriched gene-expression module.

Prior studies by our group performed on a subset of patients included in this study demonstrated that a gain-in-function mutation in DNA methyltransferase 3A (*DNMT3A;* heterozygous A/C genotype) is significantly associated with reduced risk of PB (14). In this study of 58 MRSA-infected subjects, there was a significant association between clinical outcomes and *DNMT3A* genotypes (Chi-Square Test, χ² = 10.42, p<0.001). Specifically, more patients with *DNMT3A* A/A genotype had PB outcomes, while those with *DNMT3A* A/C genotype had RB outcomes, indicating a genotype-specific relationship with clinical outcomes (**Table 1**). These findings confirm and extend our previous findings that *DNMT3A* genotype is associated with MRSA clinical outcome (14). Therefore, to assess potential relationships between *DNMT3A* and transcriptomic profiles, GE modules were compared between patients with *DNMT3A* A/A vs. A/C genotypes. Logistic regression was used to identify GE modules significantly associated with host *DNMT3A* genotype (p<0.001) GE module ME7, containing 59 genes, was significantly upregulated in patients with the *DNMT3A* A/C genotype (**Figure 2A**). Comparative expression heatmap of GE module ME7 across all subjects is shown in **Supplementary Figure S3** and **Supplementary Table S4.**

To understand the potential function of co-expressed genes in ME7, we again performed pathway enrichment analysis using ShinyGO. Pathway enrichment analysis of the ME7 module genes indicated they function in regulation of B cell receptor signaling pathway and B cell proliferation, activation, and differentiation (**Figure 2B**, **Table 3**). Graphical representation of enriched biological processes and pathways in GE modules ME7 are shown in **Figure 2B**. Significantly enriched biological processes are highlighted as an interactive clustering tree using ShinyGO (**Figure 2C**). Notably, biological processes for B cell signaling in ME7 clustered and contain key genes in B cell signaling such as *CD19, CD79A, CD79B*, *CD22*, and *FCRLA* (**Table 3**).

**Table 3:** Pathway enrichment analysis of GE module ME7 (Top ten enriched pathways are shown).

Enrichment FDR	nGenes	Pathway Genes	Fold Enrichment	Pathway	Genes
1.34E-06	5	28	83.07580175	Reg. of B cell receptor signaling pathway	*CD22, STAP1, BLK, FCRL3, CD19*
1.70E-09	8	67	55.54919281	B cell receptor signaling pathway	*CD79B, CD22, STAP1, CD79A, BLK, MS4A1, FCRL3, CD19*
7.11E-05	5	69	33.71191955	Reg. of antigen receptor-mediated signaling pathway	*CD22, STAP1, BLK, FCRL3, CD19*
1.23E-06	7	102	31.92717087	B cell proliferation	*CD22, CD79A, BLK, MS4A1, TNFRSF13C, FCRL3, CD19*
0.000880279	4	67	27.77459641	Reg. of B cell proliferation	*CD22, BLK, TNFRSF13C, FCRL3*
0.000773527	5	130	17.89324961	Reg. of B cell activation	*CD22, BLK, TNFRSF13C, FCRL3, CD19*
0.001073037	5	149	15.61156006	B cell differentiation	*CD79B, CD79A, MS4A1, FCRL3, CD19*
1.61E-06	9	271	15.45025981	B cell activation	*CD79B, CD22, CD79A, BLK, MS4A1, TNFRSF13C, FCRL3, FCRL1, CD19*
7.11E-05	8	326	11.4165519	Antigen receptor-mediated signaling pathway	*CD79B, CD22, STAP1, CD79A, BLK, MS4A1, FCRL3, CD19*
0.000803056	7	357	9.12204882	Lymphocyte proliferation	*CD22, CD79A, BLK, MS4A1, TNFRSF13C, FCRL3, CD19*

### 3.4 Identification of hub genes associated with PB and RB clinical outcomes

**Figure 3:**
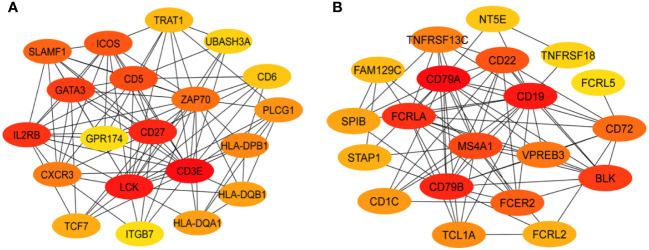
Protein-protein interaction networks identify key T and B cell immune response hub genes connected to MRSA outcome. Identification of the top 20 hub genes in ME2 (T-cell, panel **(A)** and ME7 (B cell, panel **(B)** enriched GE modules with a higher degree of connectivity. Hub genes were identified from the gene-gene interaction network using maximal clique centrality (MCC) algorithm. Edges represent the gene-gene associations. The red nodes represent genes with a high MCC sores (highly essential), uclie the yellow node represent genes with a low MCC score (less essential) by using Cytoscape software: cytoHubba plug−in.

To identify the genes most predominant in the function of each GE module, we generated PPI networks using the STRING database and CytoScape platform (32–34). We identified the hub genes within GE modules ME2 and ME7, which were significantly associated with PB or RB. For GE module ME2, a PPI network was created based on ME2 co-expressed genes (PPI enrichment p-value<1.0e-16) resulting in 89 nodes and 134 edges (**Supplementary Figure S4**). This strategy corroborated enrichment of genes involved in T cell function. The MCC algorithm identified the top 20 hub genes involved in T cell signaling and immunologically related pathways (**Figure 3A**, **Supplementary Table S5**).

**Figure 4:**
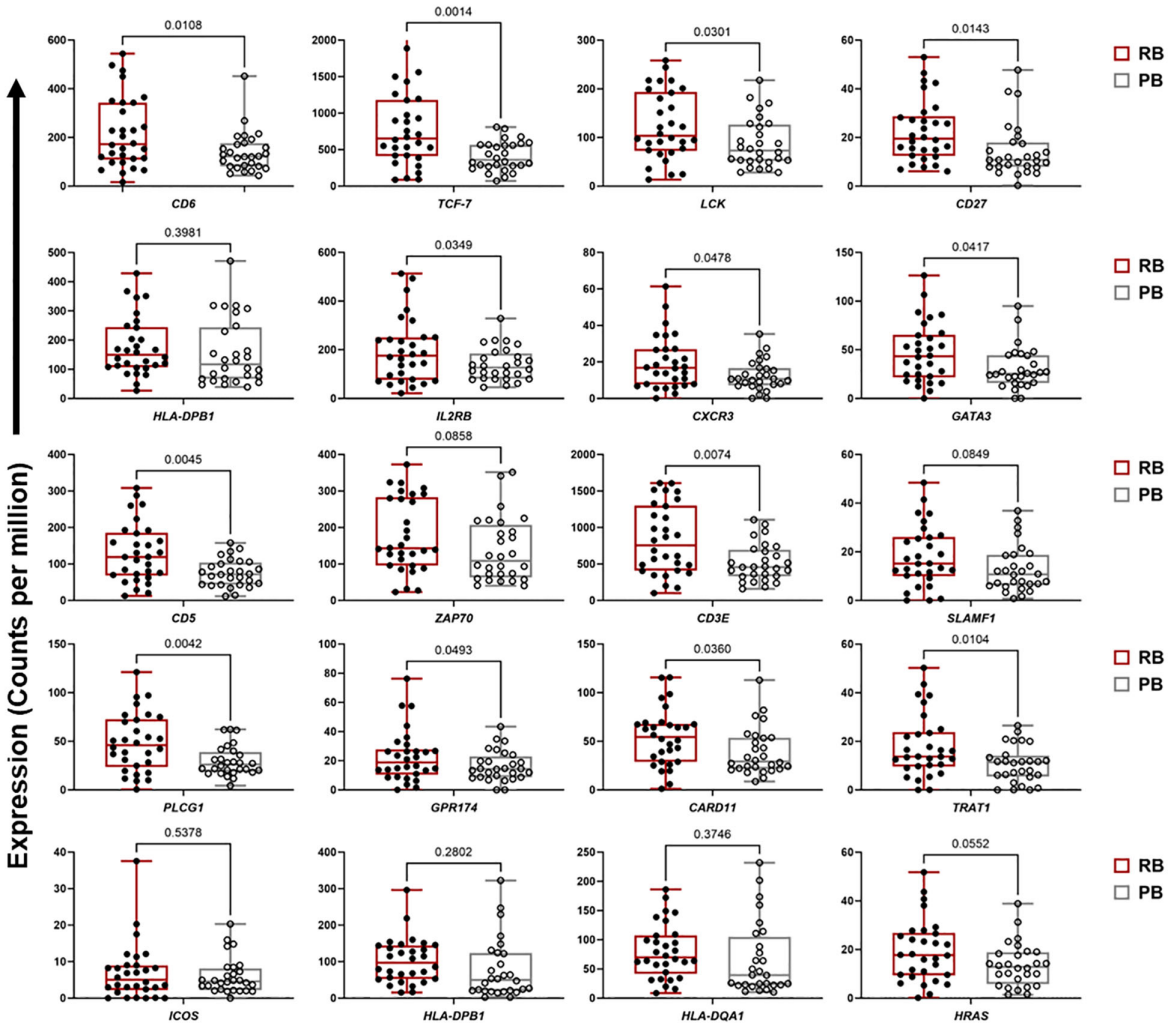
T cell signaling hub genes are upregulated in resolving vs persisting MRSA bacteremia. Boxplots of gene expression levels of the top 20 T cell signaling hub genes from ME2 in RB (n=30) vs PB (n=28) MRSA infection. Expression values are given as counts per million (CPM). p-values are based on one-sided paired t-tests.

**Figure 5:**
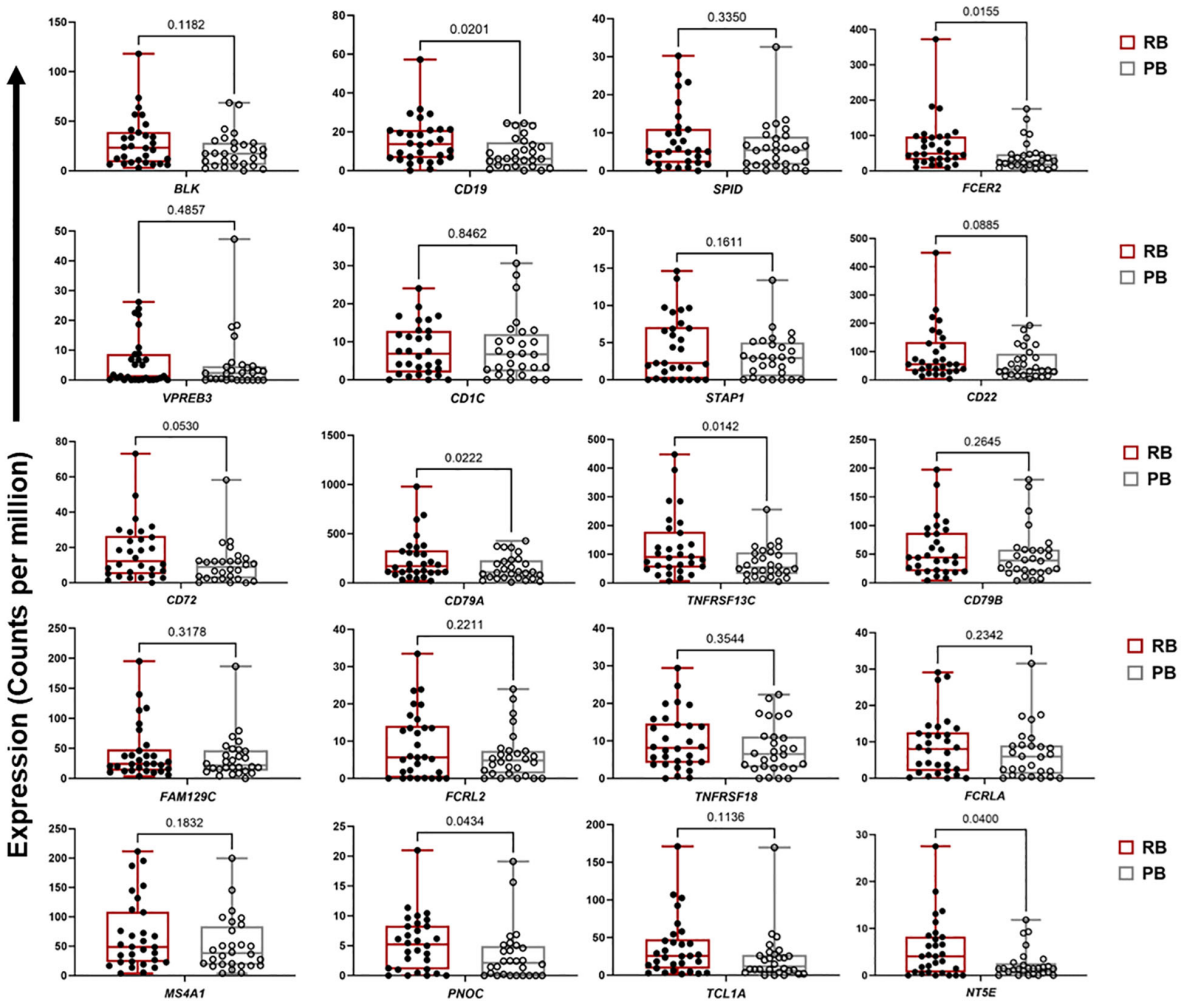
Expression of B cell signaling hub genes are downregulated in persistent MRSA bacteremia. Boxplots of gene expression levels of top 20 B cell signaling hub genes from ME7 in two clinical outcome of RB (n=30) or PB (n=28) MRSA infection. Expression values are given as counts per million (CPM). p-values are based on one-sided paired t-tests.

For ME7, which was upregulated in patients carrying the *DNMT3A* A/C genotype, a PPI network was created based on 59 co-expressed genes. The identified network contained 52 nodes and 81 edges (PPI enrichment p-value<1.0e-16) (**Supplementary Figure S5**). The MCC method identified 20 hub genes with a high degree of connectivity and function in B cell signaling pathways (**Figure 3B**, **Supplementary Table S6**). Fourteen of the 20 genes involved in T cell function were significantly upregulated in RB compared to PB (p<0.05) (**Figure 4**). Seven of the 20 B cell hub genes were significantly upregulated in RB compared to PB outcomes (p<0.05) (**Figure 5**).

### 3.5 Integration of transcriptomic, IL-10 cytokine and genotypic correlates of outcomes

**Figure 6:**
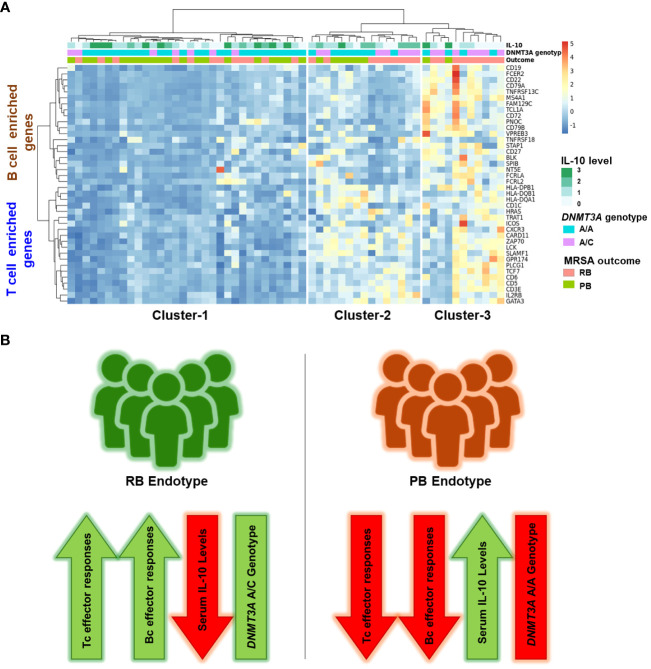
Enrichment of T cell and B cell hub genes, *DNMT3A* A/C genotype and low IL-10 cytokine concentration converge in resolving MRSA bacteremia. **(A)** Heatmap of hub genes enriched in T cell and B cell signaling (Y-axis) and Cluster number (X-axis). Clustering was performed using Clustering_method =“ward.D2”, Clustering_distance_cols =“euclidean”. Cytokine data: IL-10 was divided into 4 groups according to plasma cytokine concentration. 0.01pg/ml: denoted as 0 category, 1-20 pg/ml: denoted as 1 category, 21-40 pg/ml: denoted as 2 category, > 41 pg/ml: denoted as 3 category. Host genotype data: Two categories A/C and A/A genotype in *DNMT3A* (DNA methyltransferase-3A), A/C heterozygous genotype in the *DNMT3A* correlating with RB outcome of MRSA infection. MRSA Outcome: RB (resolvers) & PB (persistent). **(B)** Two clinical outcomes in methicillin-resistant SAB. Schematic representation of composite immune signatures in RB vs. PB endotypes of MRSA bacteremia.

The cytokine IL-10 is associated as being significantly elevated in the serum of patients with poor clinical outcome in SAB (35, 36). We previously extended this knowledge, showing that PB outcome is associated with the *DNM3TA* A/A genotype and correlated with significantly higher IL-10 levels as compared to RB outcome (14). To better understand the mechanism linking IL-10 levels to outcomes in MRSA bacteremia, we determined the relationship between identified hub genes in the B and T cell enriched signaling modules (ME7 and ME2 respectively), IL-10 cytokine levels and *DNMT3A* genotype (**Figure 6**). Based on previous findings (37), we divided IL-10 levels into 4 categories: 0.01 pg/mL (category 0), 1-20 pg/mL (category 1), 21-40 pg/mL (category 2) and >41 pg/mL (category 3) (**Supplementary Table S7**). Patients stratified into 3 clusters: Cluster 1 (n=32), Cluster 2 (n=15) and Cluster 3 (n=11) (**Figure 6A**). Binomial logistic regression was used to determine the relationship for membership of each cluster with *DNMT3A* genotype, gene expression and IL-10 cytokine level. Cluster-1 patients were associated with *DNMT3A* A/A genotype (p=0.016, Odds Ratio [OR] = 1.41, 95% confidence interval [CI] = 1.09-1.82) and expressed high IL-10 levels (p=0.181, OR = 1.09, CI = 0.96-1.22). Cluster-2 contained patients with both RB (60%) and PB (40%) outcomes. Patients in cluster-2 had lower expression of genes from the B cell enriched module compared to RB-enriched cluster-3 (p=0.382x10^-8^, linear regression coefficient = -5.544), but similar expression of genes from the T cell enriched module (p=0.0359, linear regression coefficient = 1.557), suggesting that RB outcome is most likely in patients with higher expression of both the B and T cell genes. Patients in Cluster-3 had the highest expression of genes from the T cell and B cell enriched GE modules and were significantly associated with RB outcome (p=0.027, OR = 1.26, CI = 1.03-1.53). Cluster-3 patients were also increased in the *DNMT3A* A/C genotype (p=0.036, OR = 1.25, CI = 1.02-1.54) and had lower IL-10 cytokine level (p=0.083, OR = 0.92, CI = 0.084-1.01). Conversely, patients in cluster-1 exhibited the lowest expression of genes from the T cell and B cell enriched GE modules and were significantly associated with PB outcome (p=0.011, OR = 1.37, CI = 1.07-1.75) (**Figure 6**).

### 3.6 Prediction of PB vs. RB outcomes in an independent MRSA bacteremia patient cohort

**Figure 7:**
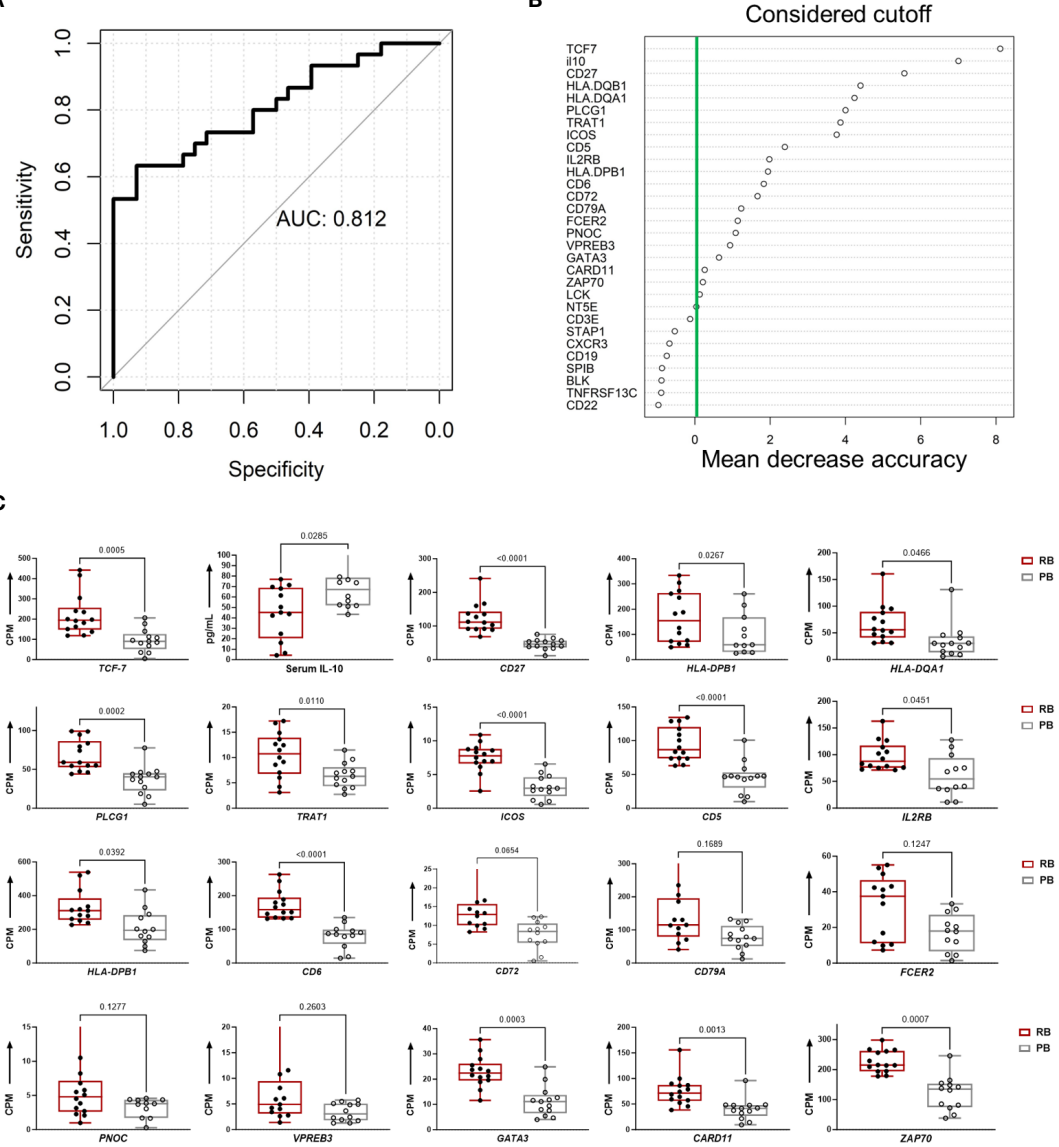
Persistent and resolving outcomes of MRSA bacteremia are predicted from hub genes and IL-10 cytokine data using random forest. **(A)** Random Forest Model for clinical outcome classification curves of receiver operating characteristics (ROC) for a random forest model using a training set cohort-1 58 subjects of MRSA infected (train set) the data. The mean AUC over 1000 random data splits is shown. **(B)** Feature importance for the random forest algorithm based on mean decrease in accuracy. The figure illustrates the feature importance values computed using the Random Forest algorithm and based on mean decrease in accuracy. The y-axis represents the importance scores, with higher values indicating greater importance for model accuracy. The green line indicates the chosen cutoff for feature importance, beyond which features are considered significant for inclusion in the model. **(C)** Top 20 classifiers in the validation cohort for predicting clinical PB vs. RB outcomes. The boxplots show the top 20 classifiers consisting of 19 T and B cell hub genes and IL-10 cytokine levels in the validation cohort. Expression values are given as counts per million (CPM) and cytokine level is in pg/mL. p-values are based on one-sided, paired t-tests.

To evaluate the predictive capacity of T and B cell hub gene expression and IL-10 cytokine levels for predicting outcome of MRSA bacteremia, we built a random forest classification model based on these 41 variables. The accuracy of this model to correctly identify clinical PB vs. RB outcomes was then compared in a distinct cohort of patients (Cohort-2). The classification accuracy of the model, evaluated by 10-fold cross-validation, was 0.812 (**Figure 7A**). The final model included the top 20 performing variables (**Figure 7B**). When this model was applied to a validation cohort (n=27), it had an 85% accuracy in predicting PB outcomes and a 30% accuracy in predicting RB outcomes (**Supplementary Table S8**). To support these findings, we compared levels of the variables included in the final model between PB and RB in the validation cohort (**Figure 7C**). This independent cohort analysis confirmed our findings that upregulation of specific B- and T cell-related genes in context of reduced IL-10 cytokine level are characteristic of RB outcome.

## 4 Discussion

In the present study, we examined the transcriptome, *DNMT3A* genotype and IL-10 proteome of whole peripheral blood to differentiate immune response pathways associated with RB versus PB outcomes in clinical MRSA bacteremia. Our central findings show upregulation of T and B cell immune response genes early during MRSA infection is associated with decreased incidence of PB in the setting of appropriate vancomycin therapy. Furthermore, transcriptional profiles correlated with lower IL-10 cytokine level and heterozygous *DNMT3A* A/C genotype, both of which have been associated with reduced risk of PB outcome in human MRSA bacteremia (14, 38).

Significant differences in transcriptional profiles were identified in PB vs. RB patients. Analysis of GE modules revealed two particularly interesting relationships: 1) T cell networks associated with PB vs. RB outcome; and 2) B cell networks associated with *DNMT3A* genotype and IL-10 cytokine level. Overall, GE modules showed significantly higher co-expression signatures of these T cell and B cell gene networks in RB as compared to PB patients. Specific hub genes within these networks included T cell activation and differentiation (**Figure 1**), and B cell receptor signaling, activation and proliferation (**Figure 2**). For example, upregulated T cell genes *CD3E, CD6, CD5*, and *CD27*, members of the T cell signaling protein family, were upregulated in RB compared to PB (**Figures 3A**, **4**). The surface proteins CD5 and CD6 modulate T cell activation in response to pathogen associated molecular patterns (PAMPs), including those found in MRSA (39, 40). Interestingly, CD27 differentiates naïve from memory T cell subsets, with greater expression on naïve T cells (41). The finding that CD27 expression is upregulated in RB patients is consistent with the notion that activation of naïve T cells is as important as that of memory T cells in controlling MRSA bacteremia. Thus, T cell receptor-mediated responses appear to play a critical role in the modulation of T cell activation, expansion, and maintenance of long-term memory important in protecting against persistent MRSA bacteremia.

Consistent with this premise, the T cell chemoattractant *CXCR3* was significantly upregulated in RB as compared to PB patients. *CXCR3* is important for trafficking and recruitment of Th1 and Th17 polarized CD4^+^ T cells in response to infection. This finding is substantiated by the fact that a mixed Th1/Th17 immune response is known to mediate clearance of MRSA infection (42, 43). *CXCR3* also recruits cytotoxic CD8^+^ T cells that contribute to clearance of infection, potentially including host cells harboring intracellular *S. aureus* (44). It should be noted that higher expression of genes does not necessarily imply greater inflammatory response. For example, many T and B regulatory cell genes are involved in modulation of immune response. Among these, IL-10 expression is characteristic of regulatory (Treg) and B10 (regulatory B or Breg) cell subsets. The fact that IL-10 levels were reduced in RB outcomes supports the concept that effector T and B cells are likely prioritized over Treg and Breg for effective clearance of MRSA from the bloodstream. Thus, taken together, the current findings support the hypothesis that appropriate expression and polarization of T and B cell responses are integral to resolution of MRSA bacteremia in the setting of vancomycin therapy.

While Th17 and Th1 T cell polarization appear important to RB outcomes, other T cell polarization pathways likely contribute as well. For instance, the transcription factors *GATA3* and *TCF7* were also identified as top hub genes in the T cell module (**Figures 3A**, **4**). *GATA3* promotes Th2 polarization and humoral immunity which protects against exotoxin-mediated complications of MRSA bacteremia (45–47). On the other hand, Th2 pathways may also modulate hyper-inflammatory immune responses to MRSA that may be detrimental to host clearance of infection (48). *TCF-7* is a transcriptional activator with critical roles in the development, differentiation, and durability of CD4^+^ and CD8^+^ T cells (49–53). This relationship with CD8^+^ T cells is of special interest, as persistent MRSA may exploit intracellular invasion to evade immune responses (54). Therefore, it is plausible that *TCF-7* upregulation contributes to immune responses that guard against persistence strategies by MRSA. Taken together, these findings suggest that specific pathways of activation and polarization of CD4^+^ and CD8^+^ T cells overall or subsets thereof are important contributors to RB outcomes.

The above results indicate crucial hallmarks of effector T cell transcriptomes in RB outcomes. By comparison, upregulation of B cell networks was identified as contributing to RB vs. PB outcomes in context of *DNMT3A* genotype and IL-10 levels. Our previous work identified a gain-in-function polymorphism in the human *DNMT3A* gene as associated with a reduced risk of PB outcomes (14). The heterozygous A/C genotype in the *DNMT3A* gene was associated with lower IL-10 cytokine level and RB outcomes as compared to the A/A genotype which correlated with PB. Hence, in the current study we sought to identify transcriptional networks corresponding to distinct *DNMT3A* genotypes using GE module analysis. Expression of GE module ME7 was significantly enriched for genes involved in B cell signaling and upregulated in patients with the *DNMT3A* A/C genotype correlating with RB (14). The top 20 hub genes in B cell function associated with the *DNMT3A* A/C genotype included: *CD1C, CD19, CD22, CD72, CD79A, CD79B, FCER2*, and *MS4A1* (**Figures 3B**, **5**). Notably, *CD19, CD22, CD72, CD79A, CD79B* have been previously implicated in B cell-mediated protection against invasive *S. aureus* infection (55). Other genes upregulated in this module include members of the B cell signaling protein family, which function in B cell receptor activation and regulation of antigen receptor-mediated signaling (**Figures 3B**, **5**). In contrast, *FCRL2*, *FCRLA* and *NT5E* are modulators of B cell response. Together, the fact that these genes were upregulated in context of the *DNMT3A* A/C genotype suggests epigenotypic regulation of DNA (methylation) modulates protective B cell responses in MRSA bacteremia.

Increased production of IL-10 has been shown in a variety of experimental models and in humans to correlate with worsened outcomes in *S. aureus* infection (56). Various investigators have also linked *DNMT3A* change-in-function polymorphisms to impaired T and B cell immune responses via dysregulated cytokine production (57–60).

The current results also suggest novel mechanistic insights underpinning this relationship. For example, the *DNMT3A* A/C genotype was associated with increased co-expression signatures of T and B cell gene networks. This correlation plausibly aligns to the A/A genotype favoring host susceptibility to PB by IL-10 modulation of protective immune responses (**Figure 6**). Further supporting this concept, our Random Forest classification model of MRSA clinical outcome using differentially expressed T and B cell hub genes and IL-10 cytokine level had good classification performance (AUC = 0.81). Notably, a subgroup of patients with RB clinical outcome carried the *DNMT3A* A/C genotype, expressed similar levels of genes in the T cell enriched module, yet had reduced expression of genes in the B cell enriched module. Conversely, a subgroup of *DNMT3A* A/A positive patients with PB expressed T cell but not B cell module genes. Therefore, the combined *DNMT3A* genotype, IL-10 level, and T and B cell hub gene signature is a more reliable predictor of RB/PB outcome (**Figure 7**). The fact that the predictive value of the composite model was affirmed in the validation cohort strengthens the potential to accurately predict RB vs. PB outcomes based on an integration of T and B cell hub gene signatures and IL-10 levels (**Figure 7**).

Beyond insights into MRSA immune evasion strategies, functions corresponding to identified T and B cell signaling pathway genes associated with PB and RB outcome in relation to IL-10 cytokine level and host *DNMT3A* genotype may also guide therapeutic development or strategies to address persistence. Several lines of evidence suggest that blocking IL-10 signaling facilitates clearance of viral infection and prevents tumor growth in animal models (61, 62). Likewise, IL-10 blockade increased clearance and abrogated hematogenous dissemination of *C. neoformans* to the brain implying this strategy has therapeutic potential in treatment of fungal infections (63). Thus, it is tempting to speculate that IL-10 blockade in patients carrying the *DNMT3A* A/A genotype would promote Th1/Th17-mediated protective immune responses and resolution of MRSA bacteremia. However, IL-10 can also act as a pro-inflammatory cytokine, particularly with respect to CD8^+^ T cell function in certain bacterial infections (64–67). Thus, the specific role and relationship of IL-10 in context of mixed Th17/Th1 responses believed essential for protection against *S. aureus* bacteremia remain to be explored.

It is important to consider the limitations of this study. The current investigation was designed to detect patterns of host immune response signals, networks and pathways that differ in PB vs. RB outcomes in MRSA bacteremia. While not designed to determine immunologic mechanisms, T and B cell signatures reported in this study were obtained and validated using three patient cohorts from a single center. Further validation with an independent cohort is an ensuing goal. Despite this limitation, using this carefully matched case-controlled study cohort, we not only identified associated transcriptional signatures of protective T and B cell gene networks, but also confirmed previous reports linking the *DNM3TA* A/C genotype and lower IL-10 cytokine level infection with PB (**Figure 6**). Future investigation to determine if these signatures exist in MRSA bacteremia patients from other geographic populations will be informative. The present studies derive from patients treated with vancomycin, the gold-standard therapy for MRSA bacteremia. One notable aspect is that not every patient with PB underwent full source control, which may contribute factors influencing the clinical outcome of PB. We are lacking specific clinical information for Minimum inhibitory concentration (MIC) values, time to therapeutic vancomycin levels, and potential missed doses in this study. However, all patients with suspected bacterial sepsis received broad spectrum antibiotic therapy that typically included vancomycin for MRSA due to high prevalence of MRSA at our institution. For this reason, there were likely no delays in vancomycin therapy. Although we are unable to confirm whether the groups were similar in terms of time to achievement of therapeutic vancomycin levels, we believe this is unlikely because vancomycin was administered based on therapeutic drug monitoring for all patients and all patients in each group were already on vancomycin when blood was drawn for transcriptomic analysis. The duration of therapy varied based on the extent of the infection, but generally ranged from two to six weeks. Further studies are also warranted to mechanistically explore whether the transcriptomic signatures associated with RB versus PB in patients treated with vancomycin holds true for other anti-infective therapies used in MRSA bacteremia. Lastly, *DNMT3A* encodes a DNA methyltransferase that plays a crucial role in epigenetic modifications which influencing gene expression patterns. The relationship between *DNMT3A*, IL-10 and adaptive immune responses involves intricate regulatory mechanisms within the immune system. Thus, altered *DNMT3A* activity may influence the expression of T and B cell genes involved in immune regulation, potentially impacting the production of multiple cytokines in response to MRSA (57, 68).

In summary, current findings underscore the importance of lymphocyte-mediated immunity for resolution of MRSA infection. The protective response was associated with genes corresponding to T and B cell functions in context of established IL-10 responses and genotypic relationships in host defense against *S. aureus*. The fact that patients with RB predominantly carried the *DMN3TA* A/C genotype is consistent with our prior findings that DNA methylation and epigenetic status governs integrated regulation of T and B cell function, and IL-10 expression shaping PB vs. RB outcomes. Finally, a major strength of this study was validation of key findings from training cohorts using an independent patient cohort analyzed in a masked manner. The implications for future studies include validating the predictive value of omics classification of PB or RB risks in larger cohorts of patients with MRSA bacteremia from diverse populations across clinical endotypes, exploring T and B cell signaling networks eliciting protective immunity to MRSA, and understanding antibiotic-specific relationships. *S. aureus* has a remarkable ability to evade host innate and adaptive immune defenses (69, 70), thus these insights may shed new light on the dynamic interplay between immune response and organism persistence strategies. The rapid onset of lymphocyte gene expression profiles linked to dysregulated host immunity in PB clinical outcomes makes this gene signature a prime target in the search for the regulatory origins of T cell and B cell dysfunction in MRSA infection (**Figure 6**). Translation of this knowledge holds promise for guiding development of novel diagnostic methods, innovative anti-infectives, vaccines and immunotherapeutic to address the challenge of persistence in *S. aureus* and perhaps other infections as well.

## Data availability statement

The data presented in the this study are deposited in the Sequence Read Archive, NCBI project repository, accession number PRJNA914756. The cytokine and clinical data that support the findings of this study are available on request from the corresponding author, [EFR].

## Ethics statement

The studies involving humans were approved by Duke University Medical Centre (DUMC). The studies were conducted in accordance with the local legislation and institutional requirements. Written informed consent for participation in this study was provided by the participants’ legal guardians/next of kin.

## Author contributions

RP: Formal analysis, Investigation, Methodology, Software, Writing – original draft, Writing – review & editing. HP: Data curation, Formal analysis, Methodology, Software, Writing – review & editing. RA: Writing – review & editing, Formal analysis. MR: Writing – review & editing, Conceptualization, Methodology, Supervision. DWG: Funding acquisition, Writing – review & editing, Formal analysis. FR: Data curation, Writing – review & editing, Funding acquisition, Methodology. LC: Data curation, Writing – review & editing. VGF: Funding acquisition, Resources, Writing – review & editing, Conceptualization, Data curation. MRY: Conceptualization, Funding acquisition, Project administration, Writing – review & editing, Resources, Supervision, Validation. EFR: Conceptualization, Funding acquisition, Project administration, Resources, Supervision, Writing – review & editing.

## Group members of MRSA Systems Immunobiology Group

MRSA Systems Immunobiology Group (MSIG) Author List: Rajesh Parmar, Richard Ahn, Liana C. Chan, Arnold S. Bayer, Yu-Ling Chang, Scott G. Filler, Vance G. Fowler, Jr., David Gjertson, Alexander Hoffmann, Felix Mba Medie, Tsuyoshi Mikkaichi, Simon Mitchell, Yan Qin, Elaine F. Reed, Maura Rossetti, Felicia Ruffin, Batu K. Sharma-Kuinkel, Katherine Sheu, Joshua Thaden, Alan J. Waring, Yan Q. Xiong, Ying Zheng, Michael R. Yeaman.

## Supplementary material

Supplementary Figure 1 Selection of the soft-thresholding powers to identified co-expression gene modules. **(A)**. The left panel showed the scale-free fit index versus soft-thresholding power. X-axis represents soft threshold (power). **(B)**. The right panel displayed the mean connectivity versus soft-thresholding power. Power 5 was chosen for which the fit index curve flattens out upon reaching a high value (> 0.9). Lowest possible power term where topology approximately fits a scale free network (on or above red horizontal line). X-axis represents soft threshold (power).

Supplementary Figure 2 Heatmap depictions of gene co-expression modules in all subjects, which are significantly associated with clinical outcome of MRSA infection. Gene expression Module ME2 includes 99 genes.

Supplementary Figure 3 Heatmap depictions of gene co-expression modules in all subjects, which are significantly associated with *DNMT3A* gene genotype of the host clinical of MRSA infection, Modules ME7 includes 59 genes.

Supplementary Figure 4 Findings of network and pathway analysis by protein-protein interaction network complex. Pathway analysis was performed with the list containing the 99 differentially co-expressed module genes associated with clinical outcome of MRSA infection. The most significant network generated was related to Th cell differentiation, & T cell signaling pathway with gene-gene interaction enrichment p-value < 1.0e-16. Here different nodes are representation of genes and edges are connections between genes, nodes colored according to functional enriched pathway they involved in.

Supplementary Figure 5 Findings of network and pathway analysis protein-protein interaction network complex. Pathway analysis was performed with the list containing the 59 DCGs associated with host’s A/C genotype of *DNMT3A* of MRSA infection. The most significant network generated was related to B signaling pathway, & regulations of immune system process with gene-gene interaction enrichment p-value < 1.0e-16. Here different nodes are representation of genes and edges are connections between genes, nodes colored according to functional enriched pathway they involved in.

Supplementary Table 1 Propensity scores in clinical cohort analysis.

Supplementary Table 2 Gene expression data normalized by CPM (counts per million).

Supplementary Table 3 Module 2 (ME2) Gene Expression Data Normalized by counts per million (CPM).

Supplementary Table 4 Module 7 (ME7) gene expression data normalized by counts per million (CPM).

Supplementary Table 5 Maximal clique centrality (MCC) scores for hub genes in module 2 (ME2).

Supplementary Table 6 Maximal clique centrality (MCC) scores for hub genes in module 7 (ME7).

Supplementary Table 7 IL-10 level (pg/ml), clinical outcome of mrsa infection, host’s *DNMT3A* genotype, and IL-10 categories.

Supplementary Table 8 Prediction scores for MRSA outcomes using the Random Forest Model.
