# Supplementary figures and images for: Integrated transcriptomic analysis reveals immune signatures distinguishing persistent versus resolving outcomes in MRSA bacteremia
Source: Front Immunol. 2024 May 23;15:1373553. doi: 10.3389/fimmu.2024.1373553 (PMC11153731; doi:10.3389/fimmu.2024.1373553)

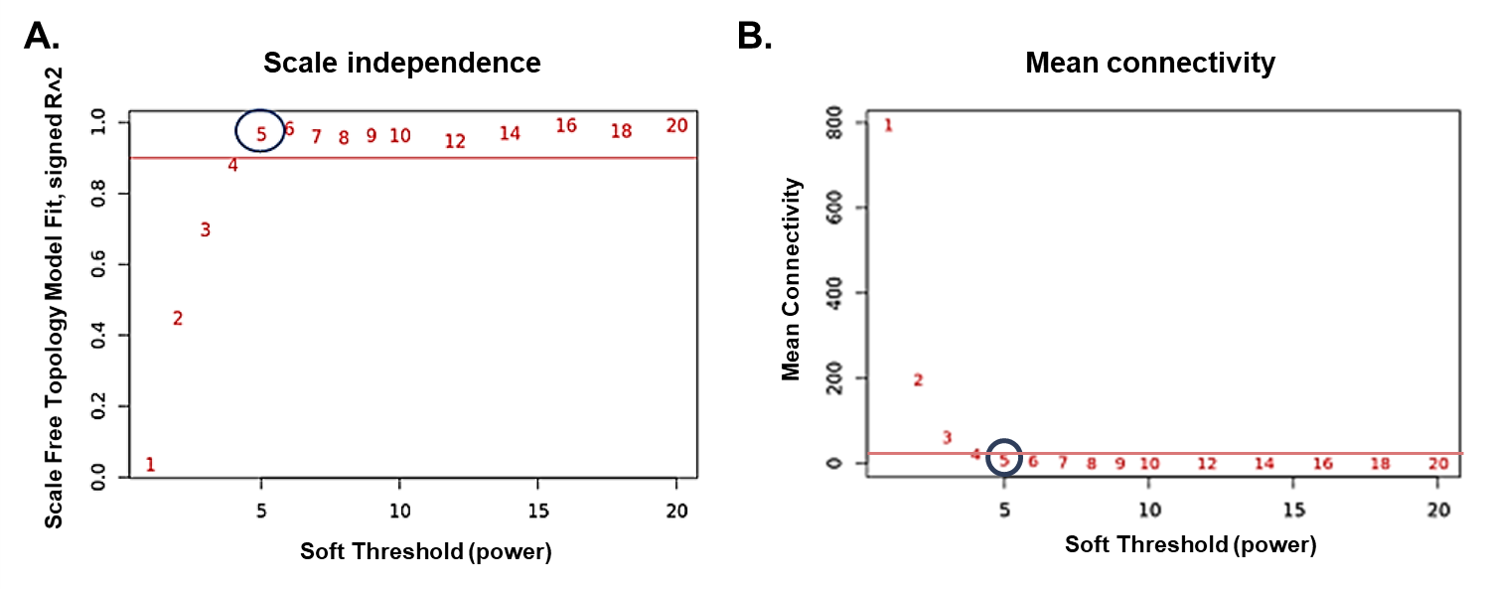

Supplement: Supplementary Figure 1 — Selection of the soft-thresholding powers to identified co-expression gene modules. (A). The left panel showed the scale-free fit index versus soft-thresholding power. X-axis represents soft threshold (power). (B). The right panel displayed the mean connectivity versus soft-thresholding power. Power 5 was chosen for which the fit index curve flattens out upon reaching a high value (> 0.9). Lowest possible power term where topology approximately fits a scale free network (on or above red horizontal line). X-axis represents soft threshold (power). [file Image_1.tif]

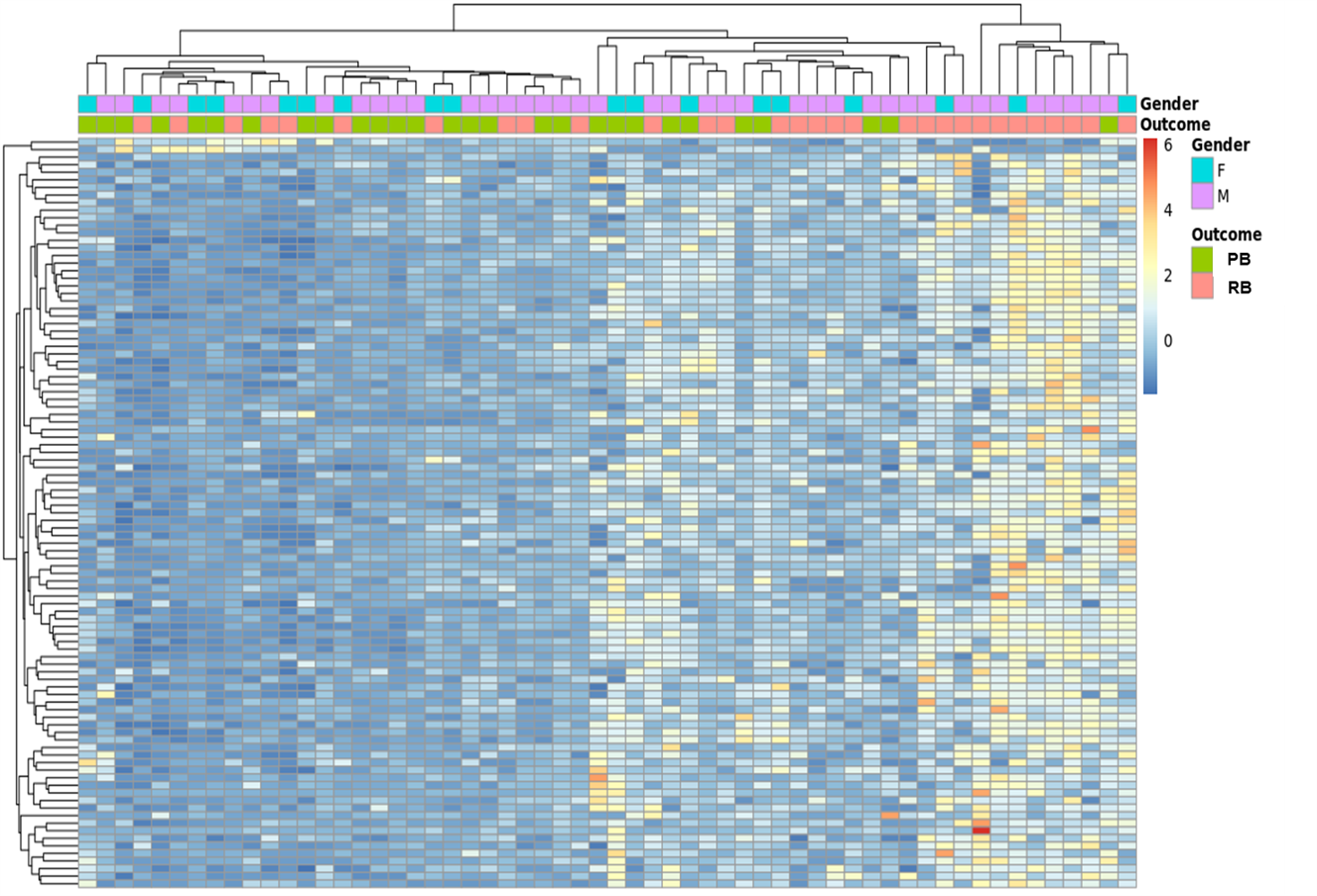

Supplement: Supplementary Figure 2 — Heatmap depictions of gene co-expression modules in all subjects, which are significantly associated with clinical outcome of MRSA infection. Gene expression Module ME2 includes 99 genes. [file Image_2.tif]

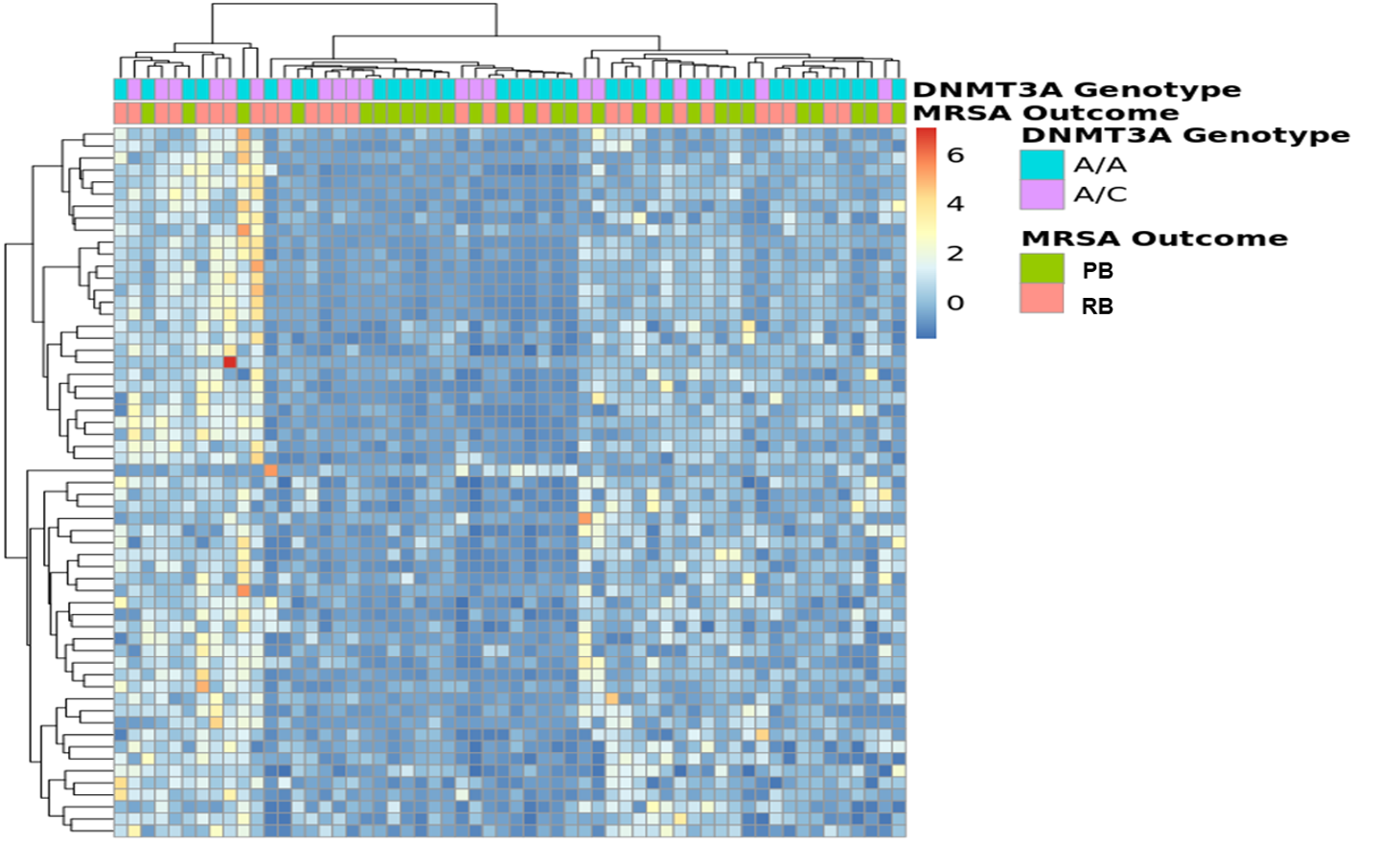

Supplement: Supplementary Figure 3 — Heatmap depictions of gene co-expression modules in all subjects, which are significantly associated with DNMT3A gene genotype of the host clinical of MRSA infection, Modules ME7 includes 59 genes. [file Image_3.tif]

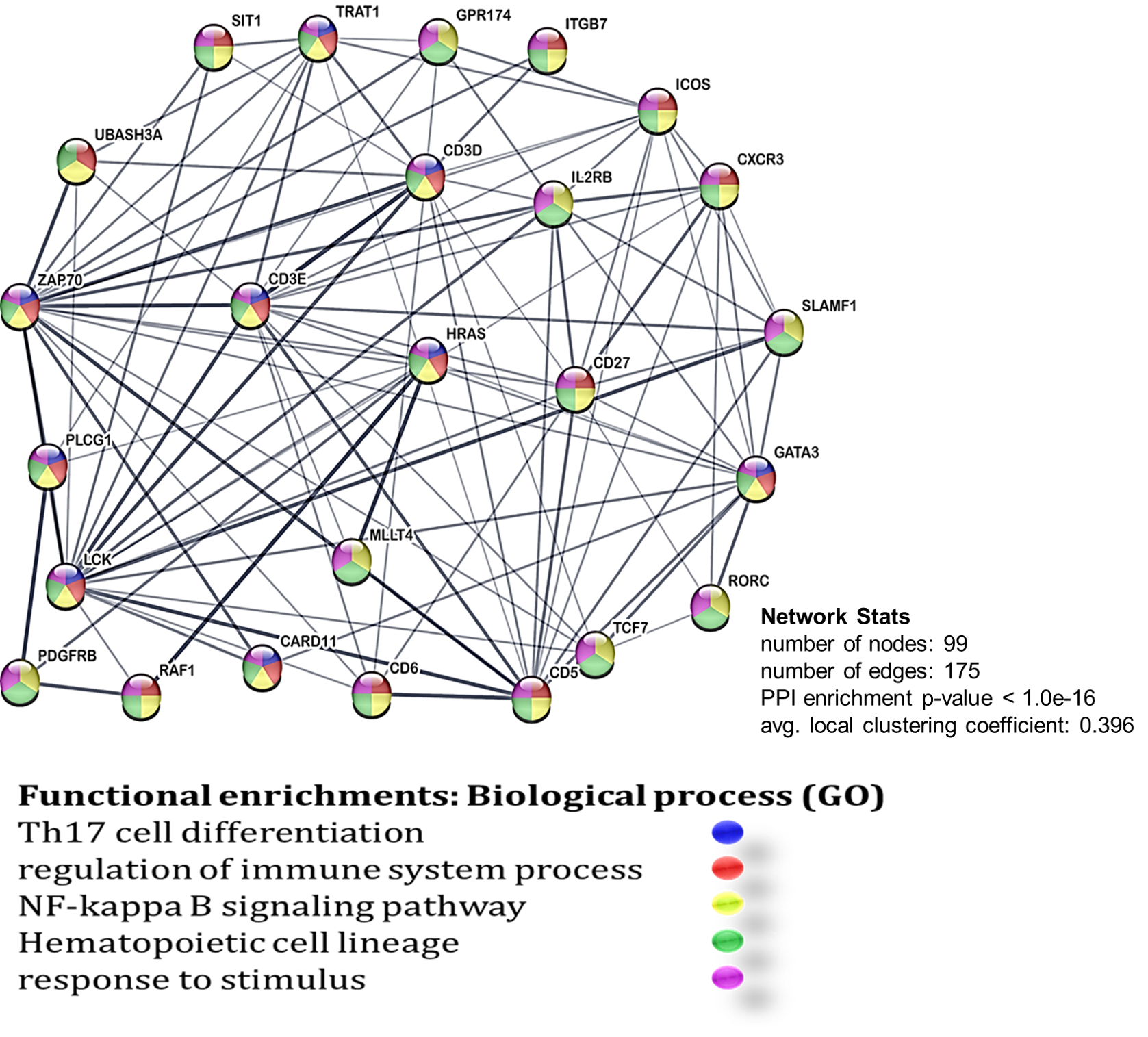

Supplement: Supplementary Figure 4 — Findings of network and pathway analysis by protein-protein interaction network complex. Pathway analysis was performed with the list containing the 99 differentially co-expressed module genes associated with clinical outcome of MRSA infection. The most significant network generated was related to Th cell differentiation, & T cell signaling pathway with gene-gene interaction enrichment p-value < 1.0e-16. Here different nodes are representation of genes and edges are connections between genes, nodes colored according to functional enriched pathway they involved in. [file Image_4.tif]

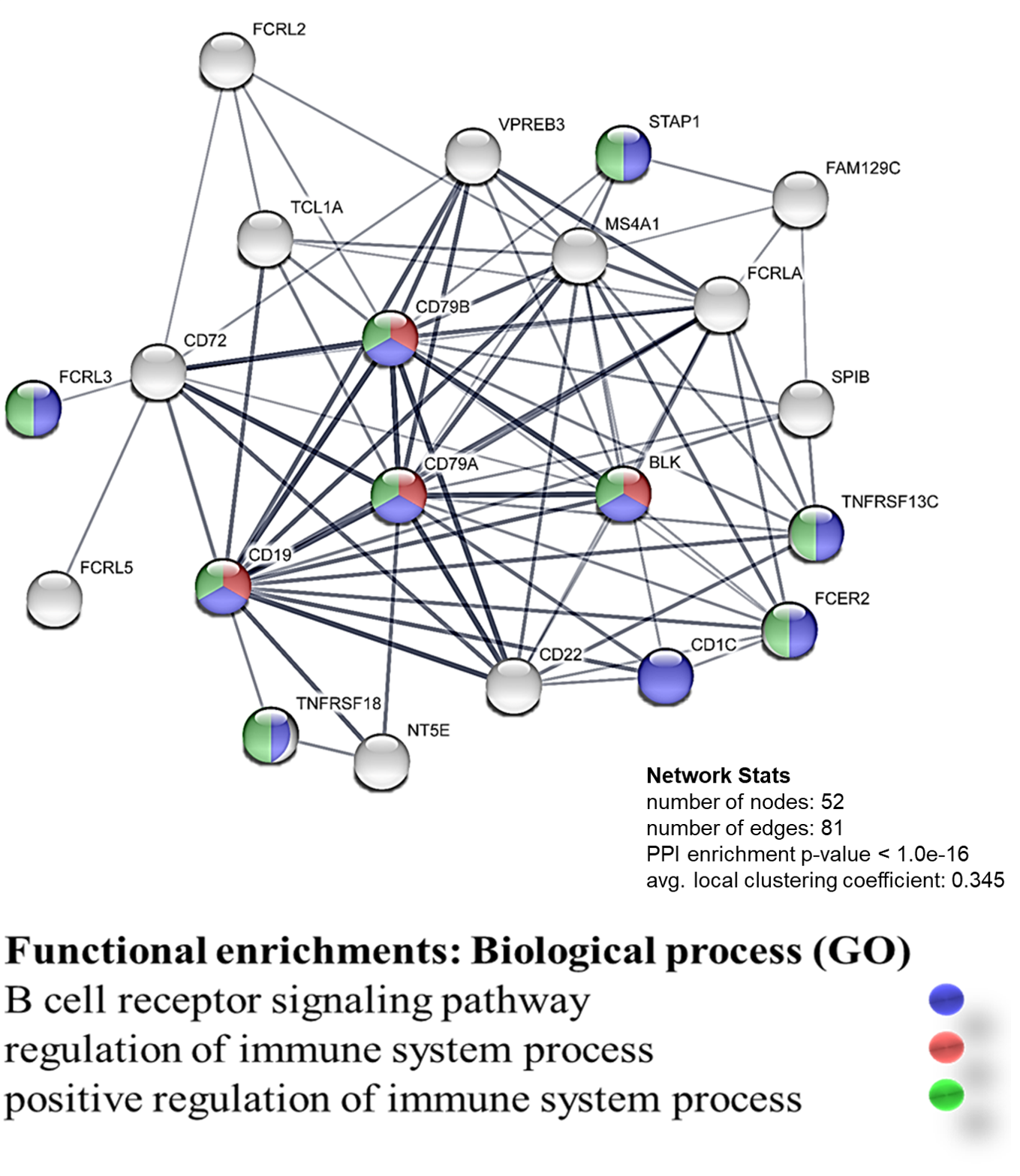

Supplement: Supplementary Figure 5 — Findings of network and pathway analysis protein-protein interaction network complex. Pathway analysis was performed with the list containing the 59 DCGs associated with host’s A/C genotype of DNMT3A of MRSA infection. The most significant network generated was related to B signaling pathway, & regulations of immune system process with gene-gene interaction enrichment p-value < 1.0e-16. Here different nodes are representation of genes and edges are connections between genes, nodes colored according to functional enriched pathway they involved in. [file Image_5.tif]
